# Comparing preference of ankle–foot stiffness in below-knee amputees and prosthetists

**DOI:** 10.1038/s41598-020-72131-2

**Published:** 2020-09-30

**Authors:** Max K. Shepherd, Elliott J. Rouse

**Affiliations:** 1 Northwestern University Department of Biomedical Engineering, The Center for Bionic Medicine Within the Shirley Ryan AbilityLab, Chicago, IL USA; 2 University of Michigan Neurobionics Lab, Ann Arbor, MI USA; 3 Department of Mechanical Engineering and Robotics Institute, University of Michigan, Ann Arbor, USA; 4 Present Address: (Google) X, Mountain View, CA USA

**Keywords:** Translational research, Rehabilitation, Biomedical engineering

## Abstract

When fitting prosthetic feet, prosthetists fuse information from their visual assessment of patient gait with the patient’s communicated perceptions and preferences. In this study, we sought to simultaneously and independently assess patient and prosthetist preference for prosthetic foot stiffness using a custom variable-stiffness prosthesis. In the first part of the experiment, seven subjects with below-knee amputation walked on the variable-stiffness prosthetic foot set to a randomized stiffness, while several prosthetist subjects simultaneously observed their gait. After each trial, the amputee subjects and prosthetist subjects indicated the change to stiffness that they would prefer (increase or decrease). This paradigm allowed us to simultaneously measure amputee subject and prosthetist subject preferences, and provided a reliability index indicating the consistency of their preferences. In the second part of the experiment, amputee subjects were instructed to communicate verbally with one prosthetist subject to arrive at a mutually preferred stiffness. On average, prosthetist subjects preferred a 26% higher stiffness than amputee subjects (*p* < 0.001), though this depended on the amputee subject (*p* < 0.001). Prosthetist subjects were also considerably less consistent than amputee subjects in their preferences (CV of 5.6% for amputee subjects, CV of 23% for prosthetist subjects; *p* = 0.014). Mutual preference seemed to be dictated by the specific patient-prosthetist dynamic, and no clear trends emerged.

## Introduction

The clinical process of matching and fitting lower-limb amputation patients with prosthetic componentry is as much art as it is science^1^, and despite decades of research on the effects of prosthetic foot mechanics on gait, there is still a gap between researcher focus and clinical practice^2–4^. While there is relative agreement about the important input variables (e.g., prosthetic foot alignment, stiffness, or energy return), answers about what to focus on or optimize are less clear. Researchers tend to base their recommendations on biomechanical analyses that are difficult to perform in a clinical setting, whereas prosthetists tend to rely on qualitative feedback from patients and visual assessment of gait to guide their decisions, which can be difficult to quantify. High quality perceptive analyses in the field of prosthetics are generally lacking. That is, it is difficult to design experiments free from subject biases, such as acquiescence bias and the placebo effect, and subjective rating scales are often coarse, unvalidated, and not easily analyzed using conventional statistical metrics^5^.

In this investigation, we focus on developing a more rigorous understanding of how people with below-knee amputation (BKA) and certified prosthetists (CP) develop and communicate preferences for prosthetic foot stiffness. Stiffness is a key determinant of overall prosthetic foot behavior; it defines how energy is absorbed and returned during collision with the ground, and how the foot provides support and ‘push-off’ in the terminal stance phase of gait. Stiffness varies by prosthetic foot model and category, and manufacturers often provide tables of recommended categories, based on patient weight and activity level. Based on discussions with clinical and industrial collaborators, we have found that these recommendations are often informally derived from internal testing with highly experienced users.

Pairing a patient with the appropriate stiffness is critical to their overall mobility, and possibly their long-term health. When paired with an inappropriately stiff foot, people with BKA may incur increased shock loading and prolonged heel contact during early stance, knee hyperextension during terminal stance, and reduced energy storage and return^6,7^. Conversely, when paired with an overly compliant foot, people with BKA may incur foot-slap in early stance and loss of anterior support in terminal stance, leading to increased quadriceps activity to maintain stability^6–8^. The exacerbation of gait abnormalities and asymmetries can lead to long-term side effects, including chronic back pain and osteopenia^9^. Researchers have sought specific biomechanical markers that indicate an appropriate stiffness, but these experiments have tended to be inconclusive, ultimately yielding tentatively issued and often contradictory guidelines^6,8,10–12^. More holistic functional outcomes, such as self-selected walking speed, metabolic cost, or balance, have also been proposed as optimizable metrics, but don’t appear particularly sensitive to foot mechanics^12–15^. Thus, while we know prescribing the right stiffness is important to overall patient quality of life, researchers have not converged on clinically viable methods for doing so.

Patient experience is dominated by outcomes that are difficult to quantify (*e.g.* socket comfort, smoothness of motion, trust in balance, or local muscle fatigue). It is unlikely that these outcomes will be perceptibly improved using more measurable variables, such as gait symmetry or metabolic cost of locomotion, as proxy. However, patients are able to sense these factors, and can often provide verbal feedback to prosthetists. This feedback may not be in the form of the intended variable, but instead in the form of the sensation, and it is the prosthetist’s responsibility to map that sensation into an appropriate change (*e.g.* a patient is unlikely to request decreased dorsiflexion stiffness, but they may complain of feeling like they are “walking uphill”^7^).

In addition to patient feedback, prosthetists can also rely on their visual assessment of patient gait. They have an internal reference, built by years of experience fitting many patients, of how a patient should ambulate with a properly chosen, well-aligned prosthesis. Prosthetists are trained to visually identify problematic gait characteristics and implement adjustments that might correct them^7,16,17^.

How should prosthetists weight their own preferences based on visual assessment against patient preferences? Complicating this question, decisions about multiple aspects of the componentry must be made with limited information about the long-term quality of the fit, and within the time constraints of a clinical visit. It is possible that their preferences align, allowing them to quickly arrive at a solution they both consider optimal. However, in instances where those preferences do not align, *repeatability* of preference may be an indicator of the validity of their internal references of ideal prosthesis behavior.

In this set of experiments, we sought to understand how stiffness preference differs between patients and prosthetists; specifically, how the independent sources of information available to a prosthetist (*i.e*. visual assessment of gait and verbal feedback from the patient) differ in both their values and their reliability. We accomplish this goal by implementing a two-alternative forced choice task, in which a BKA subject walks on a variable-stiffness prosthetic ankle set to an unknown stiffness level, and both the observing prosthetist subjects and the BKA subject silently report the directional change to stiffness they would prefer. Following these trials, we conducted a subsequent set of trials where communication between the BKA subject and a prosthetist subject was permitted, to see if mutual preference is more strongly biased towards either individual preference of the patient or prosthetist.

## Methods

Seven certified prosthetist (CP) subjects were recruited from the Shirley Ryan AbilityLab via word-of-mouth, and seven high-activity (K3-K4) subjects with below-knee amputation (BKA) were recruited through the facility’s prosthetics and orthotics clinic. Further details for the BKA subjects are presented in the Appendix. The study consisted of a complete 4 × 4 block and an incomplete 3 × 3 block, in which multiple prosthetist subjects were present for each BKA subject visit, and each prosthetist subject observed at least two BKA subjects. The split experimental blocks were chosen out of necessity, due to the difficulty in scheduling many prosthetists to be available simultaneously. Prior to the study, prosthetist subjects completed a questionnaire designed to elucidate the importance of prosthesis stiffness in clinical decision-making. This study was approved by the Northwestern University Institutional Review Board, and the study was carried out in accordance with the regulations and guidelines of the Northwestern University Institutional Review Board. Written informed consent was obtained by all BKA subjects and prosthetist subjects prior to participating in the experiment, as was written informed consent to publish their images.

A quasi-passive ankle prosthesis capable of continuous stiffness variation was used to modify ankle stiffness between trials^18^. This experimental ankle–foot consists of a rigid footplate (23.5 cm or 25.3 cm based on subject height) with attached crepe shoe material, and a rotating ankle joint. To change ankle stiffness, a small motor in the keel repositions the fulcrum of a propped cantilever spring embedded in the structure^18–20^. Notably, the torque–angle relationship is linear, and plantarflexion stiffness is 33% of dorsiflexion stiffness^19^. The experimenter commanded stiffness changes to the prosthesis wirelessly. To mask auditory clues of the magnitude and direction of stiffness changes, the motor controller separated the movement of the moving fulcrum into multiple jogs. A small degree of accuracy may be lost in simulating the mechanics of prosthetic feet with a rigid foot and dynamic ankle, but this approach reduces the complicated mechanical behavior of prosthetic feet to a single controllable, reproducible, and reportable variable: the angular stiffness of the ankle joint.

### BKA subject familiarization

A prosthetist on the research team (not included as a subject in the study) attached and aligned the variable-stiffness ankle–foot to the BKA subject’s customary socket. After familiarization with the prosthesis set to a stiffness based on a previously found weight-normalized mean preferred stiffness^19^, the subject practiced walking on the prosthesis set to stiffness levels covering a range of 66–150% of the starting stiffness. During practice, the experimenter indicated the directionality of the changes to stiffness, to educate subjects on the relationship between changes to stiffness and the associated sensations. Subjects typically walked on each stiffness level for a couple minutes, until they were comfortable and ready to try a new stiffness. After the subject practiced walking at a range of stiffnesses, the subject provided verbal feedback regarding their preferred stiffness, which then served as the reference stiffness for the experiment. This process gave the subject approximately 30 min of time walking on the prosthesis.

### Prosthetist familiarization

Before the trials began, prosthetist subjects observed the BKA subject walking at several stiffness levels, spanning the range of levels to be tested. The experimenter described the direction of the stiffness changes, and indicated the highest and lowest stiffness. Prosthetist subjects were instructed not to communicate with one another. In two of the familiarization sessions, the range of stiffness levels to be tested was shifted upwards based on the prosthetists’ request. Prosthetist subjects were informed that alignment could not be changed either before the experiment began or between trials.

**Figure 1:**
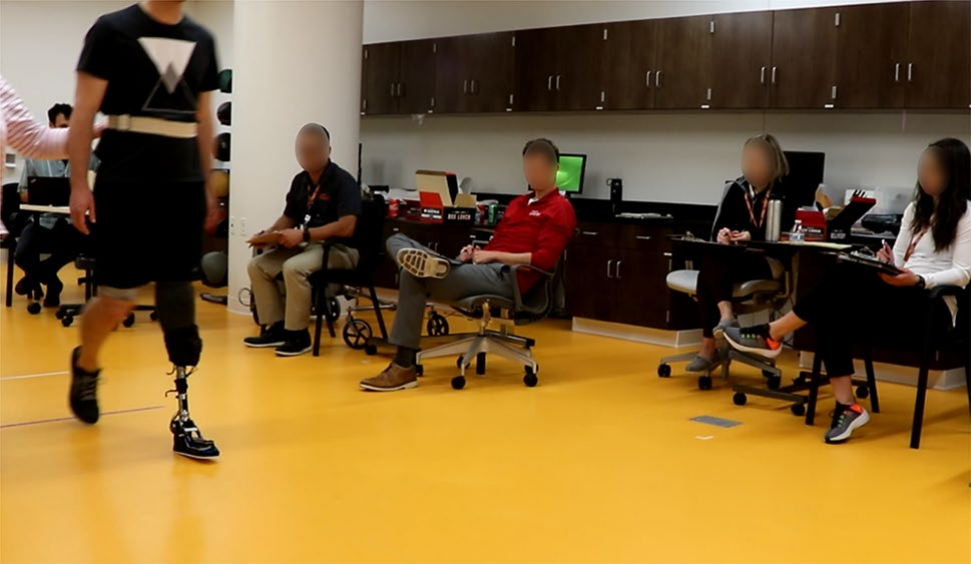
Several CP subjects assessing a BKA subject wearing the Variable-Stiffness Prosthetic Ankle–Foot.

For all trials, prosthetist subjects sat with a sagittal view of a 10 m walkway (Fig. 1). Trials consisted of the BKA subject walking down and back along the walkway, at a self-selected pace, with stiffness held constant throughout the trial. Stiffness was only changed between trials, while the BKA subject was standing still. A researcher walked on the outside of the subject, providing safety through a gait belt.

### Part 1: Identification of prosthetist and patient preferred stiffness

The first part of the experiment consisted of 39 trials for each BKA subject, with 13 stiffness levels tested three times each. The stiffness levels were logarithmically spaced around the reference stiffness, with a 7% difference between levels, and the tested range spanning 66–150% of the reference stiffness. The order of stimuli was random and determined prior to the experiment, and all subjects were informed that their responses did not affect the subsequent stimuli.

At the end of each trial, the BKA subject and prosthetist subjects indicated the directionality of the change they would like to make to prosthetic ankle–foot stiffness. Prosthetist subjects were given a score sheet on a clipboard, and after each trial they drew a small arrow to indicate whether they would recommend stiffness to be increased or decreased. Prosthetist subjects were encouraged (but not required) to write comments after each trial, describing what visual cues prompted them to select their answer. The BKA subjects indicated their preference by pointing to either an up-facing or down-facing arrow on a sheet placed on the experimenter’s desk, shielded from the view of the prosthetists. Since plantarflexion and dorsiflexion stiffness could not be independently varied, prosthetists were instructed to base their responses primarily on mid- and late-stance (dorsiflexion stiffness) in the event of competing preferences. Each trial took approximately 15 s, with an additional ~ 15 s between trials for the stiffness to be changed and for prosthetists to write comments.

To obtain a better estimate of the BKA subject’s consistency of preference, the subject completed an additional 14–23 trials after the 39 trials were completed. These trials were drawn from seven logarithmically-spaced levels, with half the spacing of the previous trials. The stimuli were centered around an estimate of the subject’s preferred stiffness data, based on the first 39 trials. During this portion, the prosthetist subjects were not in the experiment room; all but one of the prosthetists had completed the day’s experiment, with the remaining prosthetist (randomly chosen prior to the experiment) briefly waiting in a separate room prior to participation in Part 2.

### Part 2: Identification of mutually preferred stiffness

In this portion of the experiment, the prosthetist subject and BKA subject were instructed to communicate to find their mutually preferred stiffness. They were encouraged to work collaboratively, as if they were fitting the BKA subject with a new prosthesis in the clinic.

To simulate the prosthetist as the final decision-maker regarding prosthesis purchasing and fitting, the prosthetist subject communicated directly to the experimenter the changes that the pair wished to be made. The prosthetist subject could indicate both the direction and magnitude of change to be made, with “large,” “moderate,” “small,” and “tiny” changes translating to changes of 28%, 18%, 7%, and 3.5% respectively. The specific magnitudes of the changes were unknown to the subjects. Trials ended when the BKA subject and prosthetist subject arrived at the ankle stiffness they considered optimal. Four trials were completed for all subjects except BKA #1 and BKA #2, who only completed three trials due to time constraints. The starting stiffness levels were block randomized to start with either the lowest or highest tested stiffness levels, with the third and fourth starting levels equal to 33% or 66% of the range.

### Data analysis

Our statistical analysis was directed by several assumptions about preference variability and decision-making^21,22^. We assume both the percept of walking at a specific stiffness level and the internal reference of preferred stiffness are subject to Gaussian noise, and that participants follow a consistent decision rule: participants will report preferring stiffness to be increased if they perceive the present stimulus to be of lower stiffness than their preferred stiffness, and vice versa.

To obtain each participant’s preferred stiffness, their raw preference data were fit with a psychometric function (cumulative normal) using maximum likelihood estimation^22^ (lapse rate set to 1%). The Point of Subjective Equality (PSE) signifies the preference (*i.e.* the stiffness at which participants were equally likely to prefer stiffness to be increased as decreased). The steepness of the cumulative normal is inversely proportional to the standard deviation of the underlying probability density function, which describes the difference between stimulus levels and their internal notion of an ideal stimulus. Normalizing this standard deviation by the preference, we describe the consistency of each participant by the coefficient of variation (CV). For an intuitive interpretation of the CV: a CV of 0.10 indicates that if the stiffness is 10% higher than their preference, the subject will respond they would prefer stiffness be decreased 84% of the time.

The additional 14–23 trials the BKA subjects completed around their preference allowed adequate estimation of their CV. To improve the estimation of the CP subjects’ consistency, each CP’s raw data was normalized by their PSE from each session, and pooled across sessions. A new cumulative normal was fit to this pooled data. While the spacing of the stimuli was logarithmic, the fitted curves are non-logarithmic to aid in interpretability.

To determine if prosthetist subjects preferred a higher or lower stiffness than BKA subjects, prosthetist preferences were normalized by the BKA preferences, and then averaged. For example: CP #2′s preferred stiffness values for BKA subjects #1–4 were normalized to the respective BKA subject’s preferred stiffness; we then describe CP #2′s overall normalized preference as the mean of these four normalized stiffnesses. The seven prosthetist subjects’ normalized preferences were then compared against *one* with a two-sided, one-sample *t*-test. BKA-specific effects on the difference between BKA and prosthetist preferences were investigated with an imbalanced one-way ANOVA, in which there were 2–4 observations for each of the seven BKA subjects (random factor). Differences in consistency (CV) between BKA subjects and prosthetist subjects were tested with a Welch’s unequal variances *t*-test. Finally, to see if either the BKA subject or prosthetist subject had a stronger impact on the mutual preference, the mean logarithmic distances between mutual preference and the individual preferences ($\left|\mathrm{log}\left(Mutual\right)- \mathrm{log}\left(CP\right)\right|$ and $\left|\mathrm{log}\left(Mutual\right)- \mathrm{log}\left(BKA\right)\right|$) were compared in a paired *t*-test.

The prosthetist subjects’ written comments were separated into nine groups corresponding to the most common comments (for example, “drop-off effect” and “loss of anterior support” are both categorized under “drop-off effect”). The few comments that did not fit in these categories were excluded. Comments were then organized by their distance from the prosthetist’s preferred stiffness for the corresponding patient. For example, if CP #2 preferred a stiffness of 500 Nm/rad for BKA #3 and they wrote “prolonged heel contact” during a trial of stiffness 600 Nm/rad, then that comment would be logged at 1.2 (600/500).

## Results

In all but two cases, the participant’s response data could be fit with a cumulative normal function, providing a preferred stiffness and reliability score. One prosthetist subject’s responses for one BKA subject had a reversed trend and could not be fit with a cumulative normal function; this data was excluded. The responses of BKA #7 were highly consistent; the test was too short (even with the extended 17 trials the subject completed at the end of Part 1) and the tested levels too coarse to accurately estimate reliability, so this subject’s CV was excluded from analysis (see Supplementary Information Fig. S1 for subject-specific cumulative normal fits).

**Figure 2:**
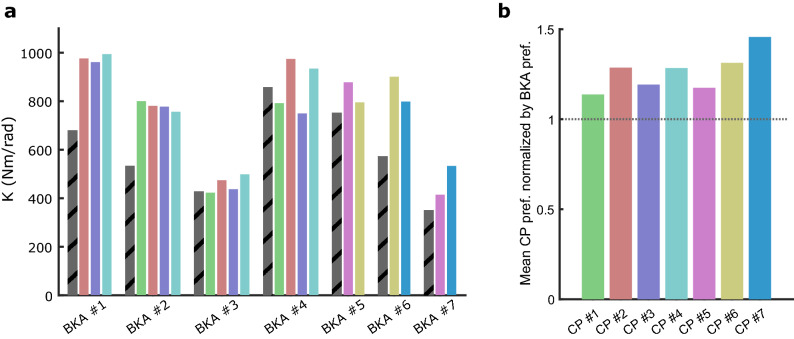
(**a**) Prosthetist preferred stiffness vs BKA preferred stiffness (striped gray bars). Each color represents a different prosthetist. (**b**) Each prosthetist subject’s preference for each BKA subject is normalized by the respective BKA subject’s preference, and averaged. The mean value is compared against 1 in a two-sided, one-way t-test (*p* < 0.001).

The prosthetist subjects and BKA subjects typically did not have the same preference (Fig. 2a). Prosthetist subjects tended to prefer a higher stiffness than BKA subjects (26% ± 11% higher), pooling each prosthetist’s normalized responses across the 2–4 patients they observed; *p* < 0.001, one-sample t-test (Fig. 2b). The difference between CP preference and BKA preference depended on the specific BKA subject (*F*_*6, 14*_ = 12.6, *p* < 0.001), with them sometimes in large disagreement (40% – 50% difference; BKA #1 and BKA #2), and sometimes in relative agreement (BKA #3 and BKA #4).

**Figure 3:**
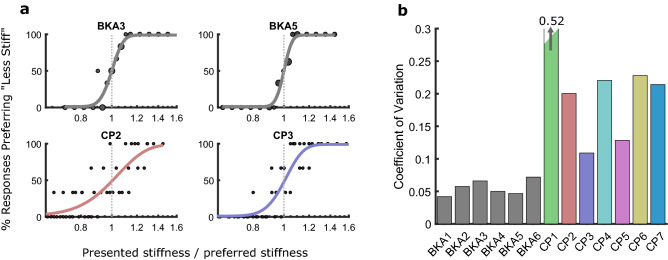
Consistency of BKA and prosthetist preferences. (**a**) Fitted psychometric functions to the preference-normalized responses of two representative BKA subjects (top) and prosthetist subjects (bottom). To improve the estimate of prosthetist reliability, their responses were pooled across sessions. Larger dots for the BKA subjects around preference indicate an increased number of trials. (**b**) Comparison of prosthetist and BKA consistencies (coefficient of variation).

Normalized preference data with fitted psychometric functions are shown in Fig. 3a for two representative BKA subjects and prosthetist subjects, and the mean CVs of each subject are shown in Fig. 3b. The BKA subjects were substantially more consistent in selecting their preference (CV: 5.6% for BKA subjects, CV: 23% for prosthetist subjects; *p* = 0.014, Welch’s *t*-test). Notably, there was high inter-prosthetist variability in consistency (SD: 14%).

**Figure 4:**
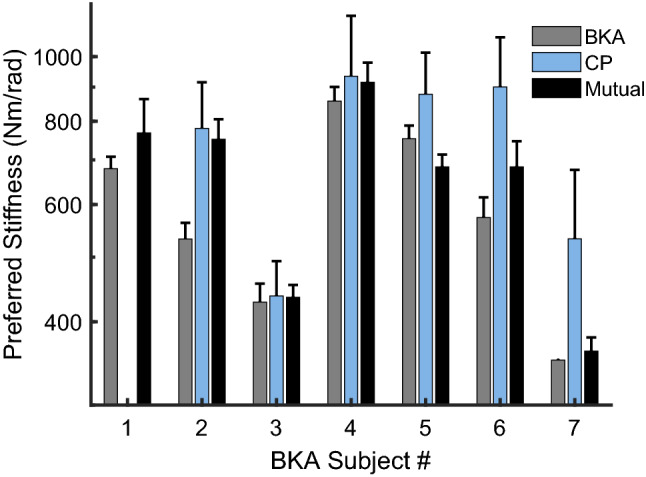
Comparison of BKA preference, prosthetist preference, and mutual preference. Error bars denote the standard deviation of the fitted cumulative normal psychometric functions for the individual preferences from Experiment 1; for the prosthetist subjects, distributions were taken from the psychometric functions fitted to their pooled (across BKA subjects) responses. Error bars for the mutual preference represent the standard deviation of the repeated preference trials from Part 2. A preference could not be determined for CP #1 (see Supplementary Information).

The nature of the interaction between BKA subject and prosthetist subject in Part 2 varied (Fig. 4). In five of the six complete sets, the mutual preference was within the range of the individual preferences. Mutual preference was not pulled consistently towards either the patient’s or prosthetist’s individual preference (*p* = 0.67). In one instance, BKA #2 was highly consistent individually but was markedly pulled towards the stiffness the prosthetist preferred, later stating that they trusted the prosthetist’s assessment, and that the prosthetist possibly knew what would be best long-term. In another session, the prosthetist deferred largely to the patient to suggest the changes, acknowledging the changes in stiffness were too small to see.

**Figure 5:**
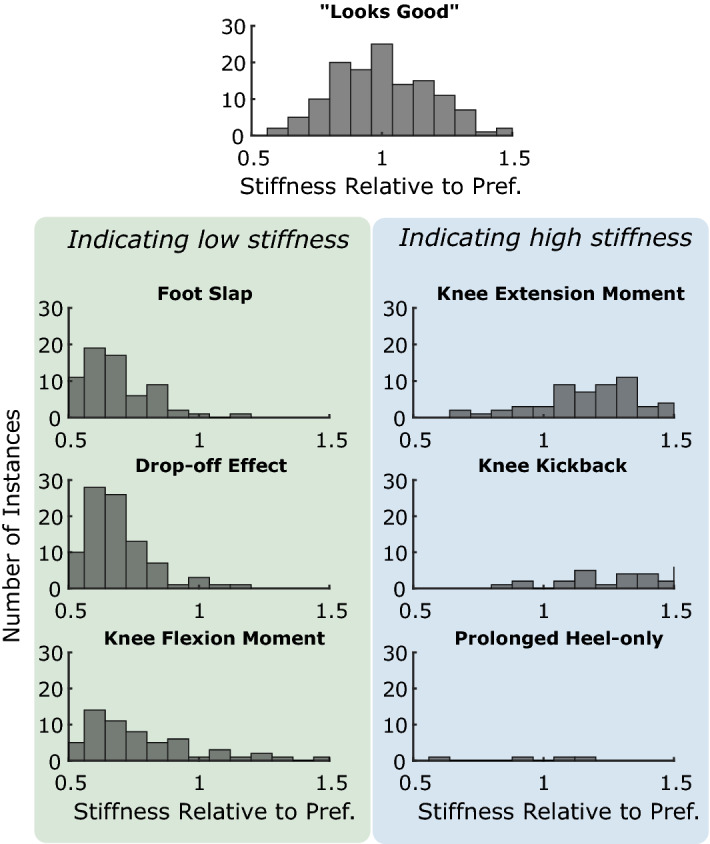
Total instances of prosthetists’ comments. Each comment is categorized as belonging to one of the above groups, or discarded. Comments are organized by their distance from the preferred stiffness. Drop off in the distributions towards the ends may due to the limits in the range of tested stiffness levels.

In their written comments, prosthetist subjects were more likely to indicate effects associated with too low of a stiffness, though this may be due in part to where their preferences fell in the tested range (Fig. 5). Of particular note, a relatively wide range of stiffness levels near the preference warranted a comment of approval; the standard deviation of this distribution is 18%.

## Discussion

To understand the complementary and competing effects of prosthetist and BKA preferences, we sought to determine their preferences for ankle–foot stiffness during level ground walking, both independently (no communication allowed), and mutually (communication allowed.) Our paradigm allowed us to simultaneously assess both absolute preferences and consistency of preferences.

One of the primary results of this study is that the prosthetist subjects preferred a higher stiffness (+ 26%) than the BKA subjects. The higher prevalence of written comments by the prosthetist subjects corresponding to a low stiffness, specifically “foot slap” and “drop-off effect,” may imply that a low stiffness may be more visually perceptible or obvious. Prosthetists may also deem a stiffness that is too low to be more detrimental than a stiffness that is too high, and thus erred on the side of an overly stiff foot. Additionally, limitations of the ankle prosthesis used in the study may have altered the experience in ways that were more easily felt by the BKA subjects than seen by the prosthetists. For example, BKA subjects may have preferred a more compliant ankle to reduce impact loading during heel-strike, or for increased energy return in late stance to help propel the higher weight of the prosthesis. Of course, in the absence of a ground truth, we cannot comment on whose preference was truly “optimal,” and would caution against over-interpretation of our result that the preferences were different.

The BKA subjects were highly consistent in selecting their preference (CV of 5.6%), with prosthetist subjects less consistent overall (CV of 23%). For a reference regarding how these sensitivities compare to foot availability, the difference between categories for the common Össur *Variflex* is approximately 11%^23^. The consistency of BKA subjects was higher in this experiment than in a previous study, in which subjects tuned the stiffness via a handheld dial while walking on a treadmill^19^; this finding suggests that perception of stiffness may be improved walking over-ground at a self-selected pace, or may be the result of fundamental differences between the method of adjustment and a two-alternative forced choice paradigm.

The higher consistency of the BKA subjects may have been due to several factors. The BKA subjects may have modified their kinetics, which are difficult to visually assess, to maintain more consistent kinematics across stiffness levels. Several prosthetist subjects commented anecdotally that certain BKA subjects in particular tended to mask changes by altering their knee kinetics (specifically noting their knee flexor and extensor activity). This may be particularly true in high-level ambulators, such as in this study, and could potentially limit the extrapolation of our results to lower-level ambulators. Natural stride-to-stride variation in gait mechanics may also mask the influence of foot stiffness to external observers, particularly given the short trial length.

Though the BKA subjects were more consistent than the prosthetist subjects, this study does not seek to quantify the quality or level of satisfaction with non-preferred stiffness levels. The wide distribution (standard deviation of 18%) of stiffness levels that prosthetist subjects commented “looked good” indicates that a large range of stiffness levels appeared acceptable. We do not have an analogous description from BKA subjects, meaning that although they have a highly specific and reliable preference, deviating from this preference may not cause a steep drop-off in satisfaction. It’s also important to note that preferences may deviate with larger accommodation times; individuals with BKA alter their gait over the course of several weeks as they adapt to a new foot^24,25^. Thus, while highly consistent within the same experimental session, their preference may change across longer time scales. These topics will be the subject of future investigations.

The results of this study indicate that allowing patients to test and learn the effects of stiffness enable them to provide reliable feedback of preference; however, without clinical tools or processes that allow patients to efficiently explore prosthesis mechanical behavior, patient feedback may be more limited. In the United States, clinics often do not carry a large assortment of test feet; instead, prosthetists order individual prosthetic feet that they believe will be most appropriate for a given patient. Accordingly, prosthetists familiarize themselves well with a small number of models^16^. Based on our questionnaire, the prosthetists in this study fit patients with one of their three most-prescribed feet 81% of the time. Similarly, and perhaps due to the impracticality of ordering multiple feet for testing purposes, the prosthetists responded that 84% of the time a patient is eligible for a new foot, they only trial a single foot.

One limitation in our study is the use of the Variable Stiffness Prosthetic Ankle–Foot to simulate changing stiffness of a conventional energy-storage-and-return prosthetic foot. In particular, the linear shape of the torque–angle response and the ratio between dorsiflexion and plantarflexion stiffness were fixed. In none of the written comments were competing effects mentioned (*e.g.,* foot-slap and knee extension moment), but one prosthetist subject mentioned competing compensations during mid- and late-stance in Part 2 of the experiment, which may have indicated that changes to alignment, foot length, or nonlinear response were needed. Another limitation is the short accommodation times, both within individual trials, and with the foot and tested range in general. The BKA subjects also received more training with the variable stiffness ankle prosthesis than the prosthetist subjects, which was done to ensure they were comfortable with the experiment and understood the tested variable, but may have given them extra time to refine their preference. Finally, the participants were not representative of their full populations: the BKA subjects participating in this study were highly active, K3–K4 ambulators, and may be more sensitive to prosthesis mechanics than lower K-level ambulators, and the prosthetist subjects all worked at the same rehabilitation hospital.

## Supplementary information

Supplementary file1
