# Supplementary material for: Comparing preference of ankle–foot stiffness in below-knee amputees and prosthetists
Source: Sci Rep. 2020 Sep 30;10:16067. doi: 10.1038/s41598-020-72131-2 (PMC7527979; doi:10.1038/s41598-020-72131-2)
Supplement: Supplementary file 1 — Supplementary file1 [file 41598_2020_72131_MOESM1_ESM.pdf]

Supplementary Information

Comparing Preference of Ankle-Foot Stiffness in Below-Knee Amputees and Prosthetists.

Max K Shepherd, PhD and Elliott J Rouse, PhD

The fitted psychometric curves for all subjects during all seven sessions are shown in Supplementary Fig. S1 As mentioned in the main text, the data from CP #1 viewing BKA #1 could not be fit with a psychometric function, and there appears to be a reversed trend. The slope from the fitted psychometric function for BKA #7 was also unreliable, and was excluded.

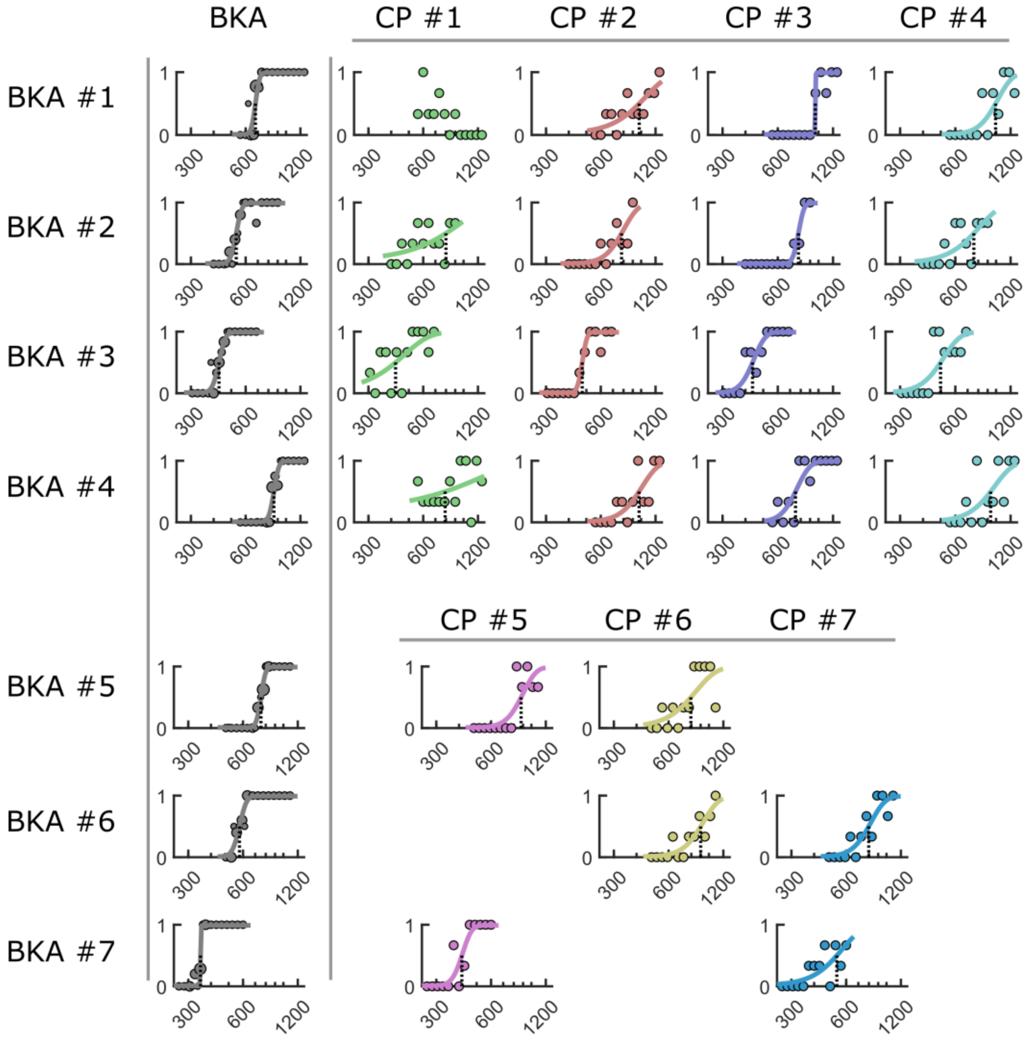

Fig. S1. Psychometric curves fit to BKA and CP response data. In each subplot, stiffness (Nm/rad) is shown on the horizontal axis, and proportion preferring lower stiffness is shown on the vertical axis. The preference is indicated with a vertical dashed line. Note that for the BKA subjects, larger circles around the preference denote more trials.

The time courses of the mutual preference trials are shown in Supplementary Fig. S2. Typically, the first trials were the longest, as the patient and prosthetist worked together for the first time.

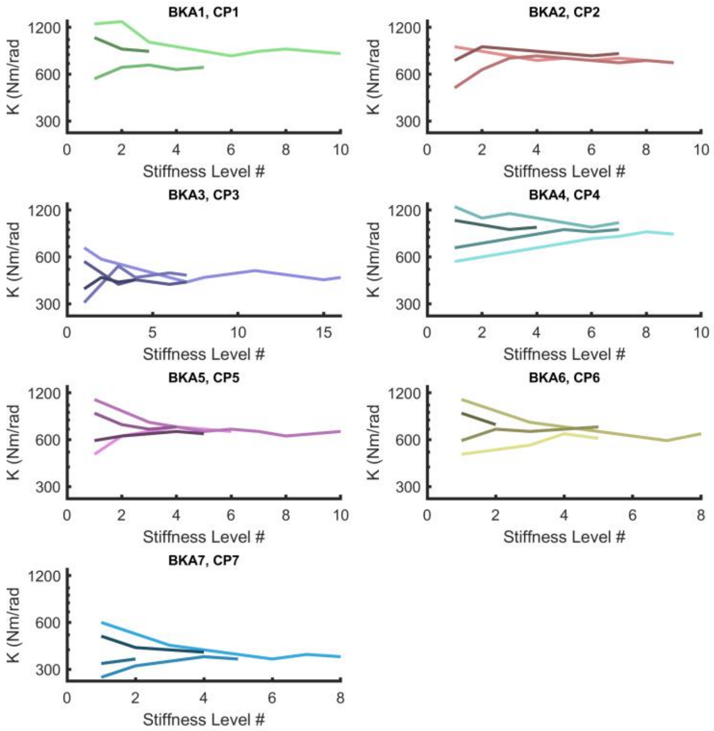

Supplementary Fig. S2. Time course of mutual preference trials. The four starting stiffness values equally spanned the tested stiffness range (0.66x – 1.5x the reference stiffness). Darker lines denote later trials.

BKA Subject information is listed in Table 1. Note: the order in the table is not linked to subject number reported in this study.

TABLE I
Subject Information

Weight (kg)	Residual Limb Length	Prescribed Prosthesis	Years since amputation
53	short	Elation, by Ossur	16
88	long	Velocity, by College Park	29
88	short	Proflex Pivot, by Ossur	5
61	short	Proflex LP, by Ossur	2
98	short	Proflex XC, by Ossur	33
90	average	Vari-flex, by Ossur	*
90	average	*	*

*Unknown
